# On Operating a Nanofiltration Membrane for Olive Mill Wastewater Purification at Sub- and Super-Boundary Conditions

**DOI:** 10.3390/membranes7030036

**Published:** 2017-07-14

**Authors:** Marco Stoller, Javier Miguel Ochando-Pulido, Robert Field

**Affiliations:** 1 Department of Chemical Engineering, University of Rome “La Sapienza”, Via Eudossiana, 18, 00184 Rome, Italy; 2 Chemical Engineering Department, University of Granada, 18071 Granada, Spain; jmochandop@ugr.es; 3 Balliol College, University of Oxford, Broad Street, Oxford OX1 3BJ, UK; robert.field@eng.ox.ac.uk

**Keywords:** membranes, membrane fouling, boundary flux, critical flux, threshold flux

## Abstract

In the last decades, membrane processes have gained a significant share of the market for wastewater purification. Although the product (i.e., purified water) is not of high added value, these processes are feasible both technically and from an economic point of view, provided the flux is relatively high and that membrane fouling is strongly inhibited. By controlling membrane fouling, the membrane may work for years without service, thus dramatically reducing operating costs and the need for membrane substitution. There is tension between operating at high permeate fluxes, which enhances fouling but reduces capital costs, and operating at lower fluxes which increases capital costs. Operating batch membrane processes leads to increased difficulties, since the feed fed to the membrane changes as a function of the recovery value. This paper is concerned with the operation of such a process. Membrane process designers should therefore avoid membrane fouling by operating membranes away from the permeate flux point where severe fouling is triggered. The design and operation of membrane purification plants is a difficult task, and the precision to properly describe the evolution of the fouling phenomenon as a function of the operating conditions is a key to success. Many reported works have reported on the control of fouling by operating below the boundary flux. On the other hand, only a few works have successfully sought to exploit super-boundary operating conditions; most super-boundary operations are reported to have led to process failures. In this work, both sub- and super-boundary operating conditions for a batch nanofiltration membrane process used for olive mill wastewater treatment were investigated. A model to identify a priori the point of transition from a sub-boundary to a super-boundary operation during a batch operation was developed, and this will provide membrane designers with a helpful tool to carefully avoid process failures.

## 1. Introduction

Fouling leads to a reduction of the permeate flux rate as a function of time, and parallel to this may lead to noticeable shortening of the lifetime of membrane modules [1]. The presence of significant fouling makes processes unsustainable from an economic point of view [2].

A precise insight to membrane fouling is currently under study. For liquid separation processes, Field et al. introduced the concept of critical flux for microfiltration, stating that there is a permeate flux below which fouling is not promptly observed [3]. Afterwards, it was possible to identify critical flux values on ultrafiltration (UF) and nanofiltration (NF) membrane systems [4]. After many years, the critical flux concept is well accepted by both scientists and engineers as a powerful membrane process optimization tool wherever critical fluxes apply [5], and un updated link with classical fouling models has been given in [6].

In the case of processing most real wastewater streams, Le Clech et al. noticed that even operations below the apparent critical flux may not guarantee zero fouling rates [7]. For the first time, these authors noticed that even when operating well below such fluxes fouling was triggered after a certain period of time, and when it grows exponentially it can significantly reduce the permeate fluxes within hours without any possibility of recovering from this situation. In other cases, it appears to be near impossible to identify critical fluxes, and only the identification of “apparent” critical points was possible. As a consequence, it becomes clear that membranes in contact with real wastewater streams behave differently from those solutions and suspensions that lead to the evolution of the critical flux concept.

In response to this issue, Field and Pearce introduced in 2011 for the first time the concept of the threshold flux, characterized by a nearly constant rate of fouling under certain circumstances [8]. The new concept was welcomed by the membrane community, since many systems that were not able to be satisfactorily described by the critical flux concept can now be described by adopting the threshold flux concept, as in the case of municipal wastewater [9].

Nevertheless, this new concept can encounter the same problems as stated previously by Le Clech et al.: even at sub-threshold flux operating conditions, after a certain period of time, severe fouling can start to build up. In subsequent years, researchers tried to explain these observations. It was found that the threshold flux depends on many factors, such as hydrodynamics, temperature, feed stream composition, and membrane surface characteristics [5]. Almost all parameters may be maintained constant during laboratory investigations, but this may not be possible with industrial streams. These parameters may change according to the adopted operating procedures (e.g., batch processing) and/or its specific nature; in particular, waste streams may sensibly change in composition as a function of time.

Finally, Stoller and Ochando-Pulido introduced the concept of boundary flux in order to merge both concepts together into one [10]. In this work, it was pointed out that the boundary flux value of a system—besides depending on the previously identified parameters—is also a direct function of time. In other words, even by maintaining fixed all input parameters of the system, in the long run, the boundary flux value will reduce and tend to zero.

The interest of this work was focused to check if the boundary flux equations may be used to predict the operating conditions which lead to severe fouling phenomena as observed previously by Le Clech and other researchers. To the knowledge of the authors, this is the first time a model will be applied to follow temporal changes in the boundary flux value from sub-boundary operating conditions to super-boundary operating conditions.

In this paper, after a brief introduction to boundary flux, the relevant equations will be used in the framework of a simulation software. The evolution of the boundary flux as a function of time and the recovery of the batch process can be calculated only by measuring initial feedstock parameters and variables. Herein, an olive mill waste stream was the chosen fouling medium. After the initial measurements, the same feedstock was used in the framework of an experimental work in order to check the fit of the simulation software output with the obtained experimental results. In this particular case, the operating conditions were chosen in such a way that operation starts in sub-boundary operating conditions, but the boundary flux changes with time (due to changing parameters). The aim was to check that the point for the onset of severe fouling was successfully predicted for this particular system.

## 2. The Boundary Flux Model

Both critical and threshold fluxes divide the operation of membranes into two regions: a lower one where either no or a small constant amount of fouling occurs, and a higher one where fouling builds up very quickly. By introducing the boundary flux, *J_b_*, in case of constant feedstock characteristics and operating conditions, the following equations may be written [11]:

d*m*/d*t* = −α; *J_p_*(t) ≤ *J_b_* (1)

d*m*/d*t* = −α + β (*J_p_*(*t*) − *J_b_*); *J_p_*(*t*) > *J_b_* (2)

where *m* is the permeability (L h^−1^ m^−2^ bar^−1^), and *J_p_* and *J_b_* are the permeate and boundary flux (L h^−2^ m^−2^), respectively. Moreover:

- α, expressed in (L h^−2^ m^−2^ bar^−1^), represents the constant permeability reduction rate suffered by the system and will be hereafter be called the subboundary fouling rate index; α is a constant, valid for all flux values.
- β, expressed in (h^−1^ bar^−1^), represents the fouling behavior in the exponential fouling regime of the system, and will be hereafter called superboundary fouling rate index; β appears not to be a constant, and changes with TMP. The chosen fitting equation used for β is equal to [11]:

β (TMP) = ζ (TMP − TMP_b_) (3)

with ζ being a dimensional fitting parameter, TMP (bar) the applied and transmembrane pressure, and TMP_b_ (bar) the applied transmembrane pressure at the boundary point.

Besides this, the model requires additional equations on mass balances and on rejection, as detailed elsewhere [12].

In sub-boundary conditions, as long as Equation (1) applies, it is possible to estimate the expected permeate flux J_p_* from a known initial value of the permeate flux *J_p_*(*t*_1_) at time *t*_1_ and same TMP value as [12]:

(4) $${J_{p}}^{*}\left(t\right)=J_{p}\left(t_{1}\right)-\alpha \int^{\text{​}}\mathrm{TMP}\;dt$$

If either Equation (1) or Equation (4) are not valid, these do not apply to the system anymore, and as a consequence, the conditions for sub-boundary flux operation are not met.

Starting from the boundary flux equation, it is possible to derive the relevant equation set for the simulation code. Most equations below use terms whose definitions are familiar to membrane technologies; they are summarized briefly here, but reported in detail elsewhere [12]. The equations include rejection calculations, material balances on the feed, concentrate and permeate stream, and fitting equations, derived from previous works and have been previously reported [12]:

(5) $$J_{b}\left(\mathrm{COD},\;t\right)=w\left(t\right)\;P_{0}-\;\alpha \;t\;P_{0}-\;\left(w\left(t\right){\;\rho }_{1}-{\;\alpha \;\rho }_{1}\;t+\;m\left(t\right)\;P_{0}\right)\;\mathrm{COD}+w\left(t\right){\;\rho }_{1}{\;\mathrm{COD}}^{2}$$

(6) $$J_{p}\left(t\right)=\;J_{p}\left(t_{1}\right)-\;\alpha \;\mathrm{TMP}\;\left(t-t_{1}\right)\;;\;J_{p}\left(t\right)\leq \;J_{b}\left(t\right)$$

(7) $$J_{p}\left(t\right)=\;J_{p}\left(t_{1}\right)-\;\frac{\alpha -\;\beta \;J_{p}\left(t_{1}\right)+\;\beta \;J_{b}}{\beta }\;\left(e^{-\beta \;\mathrm{TMP}\;\left(t-\;t_{1}\right)}-1\right);\;J_{p}\left(t\right)>\;J_{b}\left(t\right)$$

(8) $$m\left(t\right)=w\left(t\right)-\;m_{1}\;\mathrm{COD}\left(t\right)$$

(9) $$\mathrm{TMP}\left(t\right)=P_{0}-\;\pi \left(t\right)=P_{0}-{\;\rho }_{1}\;\mathrm{COD}\left(t\right)$$

(10) $$R\left(t\right)={\;\sigma }_{\mathrm{COD}}\;\frac{\mathrm{TMP}\left(t\right)}{\mathrm{TMP}\left(t\right)+\;\gamma }$$

(11) $${\mathrm{COD}}_{p}\left(t\right)=\left(1-R\left(t\right)\right)\;\mathrm{COD}\left(t\right)$$

(12) $${\mathrm{COD}}_{c}\left(t\right)=R\;\mathrm{COD}\left(t\right)$$

(13) $$F_{c}\left(t\right)=\;F_{f}-\;F_{p}\left(t\right)=F_{f}-\;A\;J_{p}\left(t\right)$$

(14) $$F_{c}\left(t\right){\;\mathrm{COD}}_{c}\left(t\right)=\;F_{f}\;\mathrm{COD}\left(t\right)-\;F_{p}\left(t\right){\;\mathrm{COD}}_{p}\left(t\right)$$

(15) $$w\left(t\right)=\;\frac{J_{p}\left(t\right)\;+\;\alpha \;t\;\mathrm{TMP}\left(t\right)\;+\;m\left(t\right){\;\rho }_{1}\;\mathrm{COD}\left(t\right)}{\mathrm{TMP}\left(t\right)}$$

where COD (mg L^−1^) is the chemical oxygen demand of the feedstock and taken as the key parameter for its characterization; *m* and *w* (L h^−1^ m^−2^ bar^−1^) are the permeability and the pure water permeability of the membrane, respectively. The terms ρ_1_ (bar L mg^−1^), m_1_ (L^2^ h^−1^ m^−2^ bar^−1^ mg^−1^), and γ (bar) are fitting parameters, *t* (h) is the operating time, σ (–) is the reflection coefficient of the membrane, R the rejection, π the osmotic pressure, and *P*_0_ the applied pressure at start of operation (*t* = 0). *A* is the membrane area (m^2^), *F* is a flow rate (L h^−1^), and the suffixes f, c, and p stand for feed, concentrate, and permeate, respectively.

At this point, particular emphasis should be devoted to Equation (5); that is, the calculation of the boundary flux value, which may change not only as a function of time t and COD, but also as a function of w. The latter parameter should not change in sub-boundary flux conditions, where almost reversible fouling occurs which can be removed during washing. The same do not apply as soon as irreversible fouling occurs, which reduces the permeability of the membrane, and consequently the relevant *w* value. To follow back this aspect, as soon as super-boundary operating conditions triggers, the value of *J_p_* as a function of time is evaluated by Equation (7). Due to the presence of β ≠ 0, this latter value is surely lower than that calculated for the same t_2_ value by Equation (6). The difference between the permeate flux values *J_p_* by Equations (6) and (7) is given by the additional formation of irreversible fouling on top of the reversible one. As a consequence, this difference is given by an irreversible reduction of the membrane permeability; that is, a corresponding and definitive change in the pure water membrane permeability as evaluated by Equation (15).

## 3. The Experimental Setup

**Figure 1:**
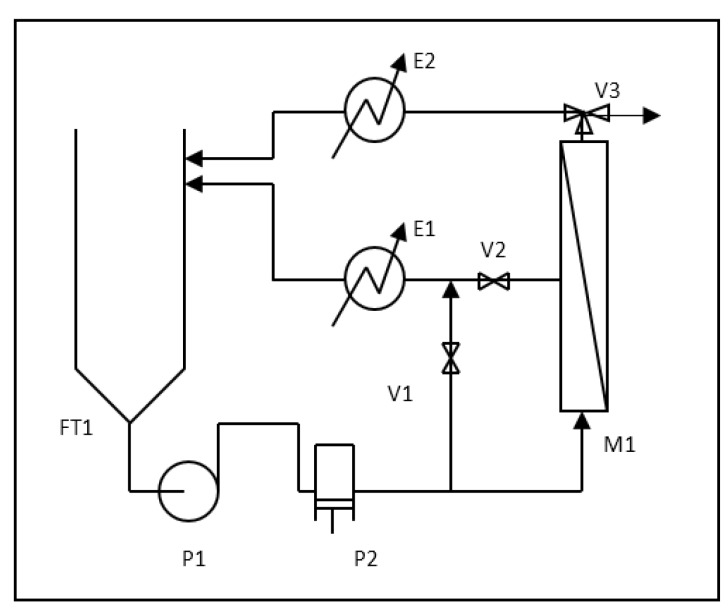
Schematic of the pilot plant. E: heat exchanger; FT: feed tank; M: membrane housing; P: pressure; V: valve.

The pilot plant that was used is shown schematically in Figure 1. The plant consists of a 100 L feed tank FT1, in which the pretreated feedstock is carried. The centrifugal booster pump P1 and the volumetric pump P2 drive the wastewater stream over the spiral-wound nanofiltration (NF) membrane supplied by Osmonics/GE Water, fitted in the housing M1, at an average flow rate equal to 600 L h^−1^. The membrane (model DK2540F) is characterized by a mean pore size value of 0.5 nm, and was used for more than 1000 h of operation time. The active membrane area of the module is equal to 2.51 m^2^, and the maximum allowable operating pressure is equal to 32 bar. Acting on the regulation valves V1 and V2, it is possible to set the desired operating pressure P over the membrane maintaining the feed flow rate constant with a precision of 0.5 bar.

Depending on use, the position of V3 can be changed and the permeate stream can be separated (batch mode) or both permeate and concentrate streams are cooled down to the feedstock temperature, mixed together, and recycled back to the feedstock, when operation at constant feedstock conditions is required and the feedstock composition is kept constant during parameter evaluation. The temperature was controlled for all experiments at the value of 20 ± 1 °C by heat exchangers E1 and E2.

A value of *w*(0) was measured before the experimental run by using bi-distilled water.

## 4. Results and Discussion

In a first stage, the characteristics of the adopted feedstock were measured and used as input values for the simulation code (see Table 1). The feedstock was an olive mill wastewater stream pre-treated by coagulation, and the characterization of the pollutant concentration was performed by COD measurements. This latter is taken as key parameter for the feedstock concentration in the model.

For other parameters (e.g., β and ζ), the relevant value can be found in other papers published by the authors [11].

**Figure 2:**
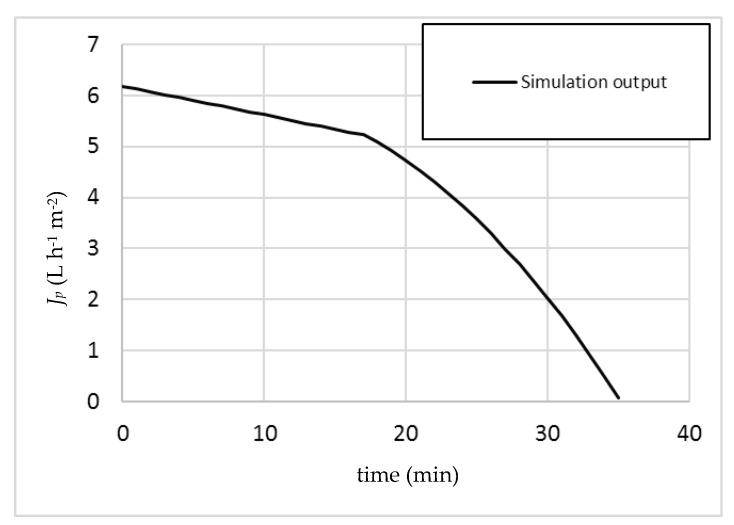
Simulation of the operation performed at 7 bar.

In this work, the boundary flux was measured by adopting the pressure cycling method proposed by Espinasse et al. [13]. Values of *J_b_* and TMP_b_ were found to be 12.18 L h^−1^ and 8 bar, respectively. In order to run the simulation, an operating pressure value must be fixed and was set equal to TMP_b_ less a safety margin of 1 bar—that is, 7 bar. The obtained output by the model is reported below (Figure 2).

It is important to run the simulation at small integration time values in order to follow the evolution of the system parameters and variables as a function of time as precisely as possible, and to maintain the validity of the used equation set. This is especially true for Equation (8), since in addition to the permeability decrease due to fouling as a function of time, the permeability value m must be also adjusted to fit the changing COD value of the feedstock.

In this study, the integration time of the simulation model was set at 5 s.

It is possible to observe that the choice to set the operating pressure below TMP_b_ appears not to be a safe choice, since at a certain point a kink appears in the plot, corresponding to the appearance of a β value in the relevant calculations, and therefore the appearance of irreversible fouling. The simulation predicts that a transition from sub-boundary to super-boundary operating conditions will occur after 16.9 min of operation.

**Figure 3:**
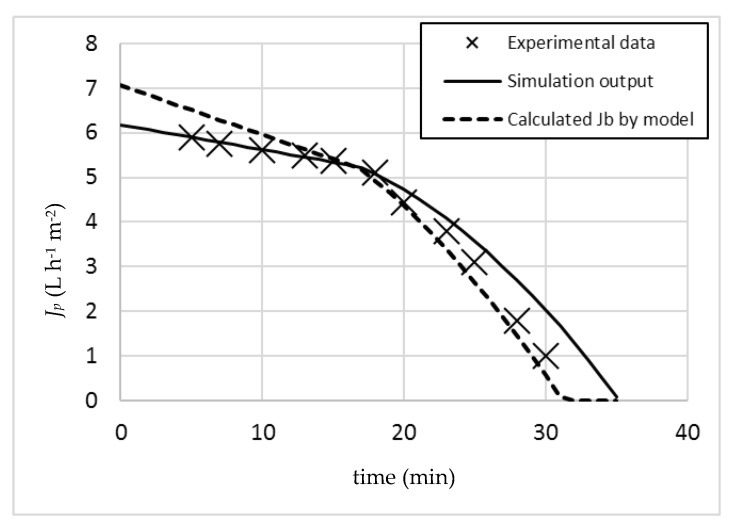
Comparison of the simulation with the obtained experimental data.

In order to check the validity of this prediction, an experimental run in batch mode was performed by maintaining the same operating pressure (7 bar). The obtained results are merged to Figure 2, resulting in Figure 3.

The experimental data showed a noticeable permeate flux reduction between 18 and 20 min of operation. In these conditions, the membrane suffers irreversible fouling, and therefore operation was stopped after 30 min without having reached the desired recovery target of 95%. By overlapping the data of the simulation model and the experimental data, the fit appears to be satisfactory.

In Figure 3, the calculated *J_b_* values by the model were plotted as well (dotted line). It is possible to observe how the kink appears as soon as the permeate profile crosses the relevant *J_b_* line. Since *J_b_* is not constant, but a function of time and operation (TMP, COD, R), this evaluation is only possible by proper model software application: indeed, *J_b_* reduces as a function of time not only due to the α value, but also due to the increase in COD given by the batch concentration effect following the increasing recovery values. As a consequence, the relevant *J_b_* values (calculated by Equation (5)) are reducing faster than *J_p_*, which is function of α only (Equation (6)). As soon as *J_b_* becomes lower than *J_p_*, Equation (6) is substituted by Equation (7). In this second part of the operation, β is no longer equal to zero, and consequently, the almost linear flux profile of *J_p_* during the first part of operation starts to assume exponential characteristics. The same applies to *J_b_*, since the value of *w*(*t*) in Equation (5) is sensibly reducing as well, due to the irreversible fouling formation: this latter value can be calculated by Equation (15), with a permeate flux value equal to that resulting from Equation (7).

The above result, showing that the transition from sub- to super boundary flux operation can be predicted in a batch operation, confirms that the set of equations given in Section 2 are capable of following the evolution of fouling with increasing concentration during the batch treatment of olive oil waste water. This was possible since this system has been completely characterized by the authors with respect to the model’s requirements. The latter is the result of much experimental efforts. As the equivalent information is not available for other waste water streams other than olive mill waste water, further validation was not possible at this stage. With regard to other systems, it is opinion of the Authors that the same methodology can be applied to those systems that follows the same expressions for fouling and the boundary flux (e.g., to Equation (5)).

## 5. Conclusions

The boundary flux concept appears to be a suitable instrument as a process operation tracking tool for batch membrane processes, given the objective of avoiding operating regions leading to irreversible fouling, provided allowance is made for the evolution of the boundary flux value.

Additional information (e.g., all parameters reported in Table 2) is mandatory in order to properly describe the system. This is particularly true for all parameters concerning the sub-boundary operating conditions, such as α, which has to be provided, together with at least one boundary flux value, in order to correctly describe the system.

However, if the pollutant concentrations change (as will naturally occur in a batch process), then even the indication of an initial α value may not be sufficient. This means that simple use of Equation (4) is not sufficient. Nevertheless, the model presented here is sufficiently comprehensive to account for these changes. All of the data necessary to set up a model in order to perform the necessary calculations have been outlined. With this model, the fouling behavior of the examined membrane process can be estimated over both short and long periods of time. The presented approach was validated for the presented case study system through experiments that passed the system from sub-boundary to super-boundary operating conditions.

In future work, the model should be applied to other systems and/or a wider range of operating conditions. The authors believe that the application will be successful. With such success, the presented methodology may be implemented into advanced control systems to give a capacity capable of avoiding adverse fouling events during long run operations.

## Figures and Tables

**Table 1:** Input values for the simulation code. COD: chemical oxygen demand.

Parameter (Units)	Value	Parameter (Units)	Value
*m*_1_ (L^2^ h^−1^ m^−2^ bar^−1^ mg^−1^)	11 × 10^−6^	α (L h^−2^ m^−2^ bar^−1^)	130 × 10^−3^
ρ_1_ (bar L mg^−1^)	0.0	ζ (h^−1^ bar^−2^)	27 × 10^−3^
*w*(0) (L h^−1^ m^−2^ bar^−1^)	1.07	COD (mg L^−1^)	17,400
σ (–)	36.80	γ (bar)	0
TMP_b_ (bar)	8		
